# The Effect of a Long-Term, Community-Based Exercise Program on Bone Mineral Density in Postmenopausal Women with Pre-Diabetes and Type 2 Diabetes

**DOI:** 10.2478/hukin-2014-0088

**Published:** 2014-11-12

**Authors:** Marieni Bello, Maria Cirilo Sousa, Gabriel Neto, Leonardo Oliveira, Ialuska Guerras, Romeu Mendes, Nelson Sousa

**Affiliations:** 1 Department of Physical Education; Federal University of Piauí, Piauí, Brazil.; 2 Department of Physical Education; Federal University of Paraíba, Paraíba, Brazil.; 3 Department of Physical Education; University Mauricio de Nassau – Paraíba, Brazil.; 4 Federal Institute of Science Education and Technology – Sector Juazeiro do Norte, Brazil.; 5 Research Center in Sport Sciences, Health Sciences and Human Development, University of Trás-os-Montes e Alto Douro, Vila Real, Portugal.

**Keywords:** bone density, fat-free mass, aging, diabetes mellitus, exercise, females

## Abstract

The aim of this study was to evaluate the impact of a community-based exercise program on bone mineral density and body composition in postmenopausal women with pre-diabetes and type 2 diabetes. Twenty postmenopausal women (aged 61.3 ± 6.0 years) with pre-diabetes and type 2 diabetes were randomly assigned to a community-based exercise program group (n=10) or a control group (n=10). The community-based exercise program was multicomponent, three days per week for 32 weeks, and included walking, resistance and aquatic exercises. Body composition and bone mineral density were measured pre and post-training by dual X-ray absorptiometry. In the exercise group significant increases were found in the ward’s triangle bone mineral density (+7.8%, p=0.043), and in fat-free mass (+2.4%, p=0.018). The findings suggest that regular multicomponent training is effective in preventing osteoporosis and sarcopenia among postmenopausal women with pre-diabetes and type 2 diabetes.

## Introduction

Osteoporosis is metabolic disorder which may result in fragile bones that are more likely to fracture (Becker et al., 2006). With aging of the population, developing safe and effective strategies to prevent osteoporosis and consequent fractures is of great importance. Its incidence is particularly high in postmenopausal women (Finkelstein et al., 2008). In addition to osteoporosis, aging also results in loss of muscle mass that accompany the age-associated decay of bone health and muscle function (Doherty, 2003).

Due to the declining estrogen levels, postmenopausal status is associated with an increased risk of pre-diabetes and/or type 2 diabetes (T2DM) (American Diabetes Association, 2013). Moreover, T2DM is associated with higher osteoporotic fracture risk (Strotmeyer et al., 2011).

Regular physical exercise has been recommended as an effective and safe nonpharmacological strategy to counter the aging-induced loss of bone mineral density (BMD). Furthermore, exercise is now considered as a key component to reduce body fat and increase fat-free mass, which results in significant improvements in functional performance (Marques et al., 2011). Walking and aquatic exercise are widely practiced by elderly women in Brazil, since they represent a safe and comfortable exercise mode for all populations at risk of falls and osteoporotic fractures (Pernambuco et al., 2013). However, the literature has shown that such exercise does not induce significant osteogenic stimuli, due to the diminished impact on bones provoked by low gravitational action in the water environment, while walking provides a modest increase in the loads on the skeleton under gravity (Gomez-Cabello et al., 2012). It is commonly accepted that resistance exercises with free weights are the best method to improve fat-free mass, and provide an osteogenic stimulus to improve and maintain BMD (Gomez-Cabello et al., 2012).

Recently, multicomponent training (MT) has been proposed as an interesting training alternative to more traditional exercise regimens, particularly due to the potential of MT to improve functional performance in older adults (Marques et al., 2011; Sousa and Mendes, 2013). MT is defined as a well-rounded program of aerobic, resistance, coordination, balance, and flexibility exercises in land and aquatic environments. To the best of our knowledge, to date, no studies have assessed the relative effectiveness of MT in post-menopausal women with pre-diabetes or T2DM with regard to their impact on BMD and body composition. Therefore, the aim of this study was to assess the effects of an 8-month program of MT on BMD and body composition in post-menopausal women with pre-diabetes and T2DM.

## Material and Methods

### Experimental Approach to the Problem

A repeated-measures design involving two groups (a training group and a control group) was used in order to determine the effectiveness of a 32-weeks community-based exercise program on BMD and body composition in post-menopausal women with pre-diabetes and T2DM. A randomized controlled study was conducted.

#### Subjects

**Figure 1:**
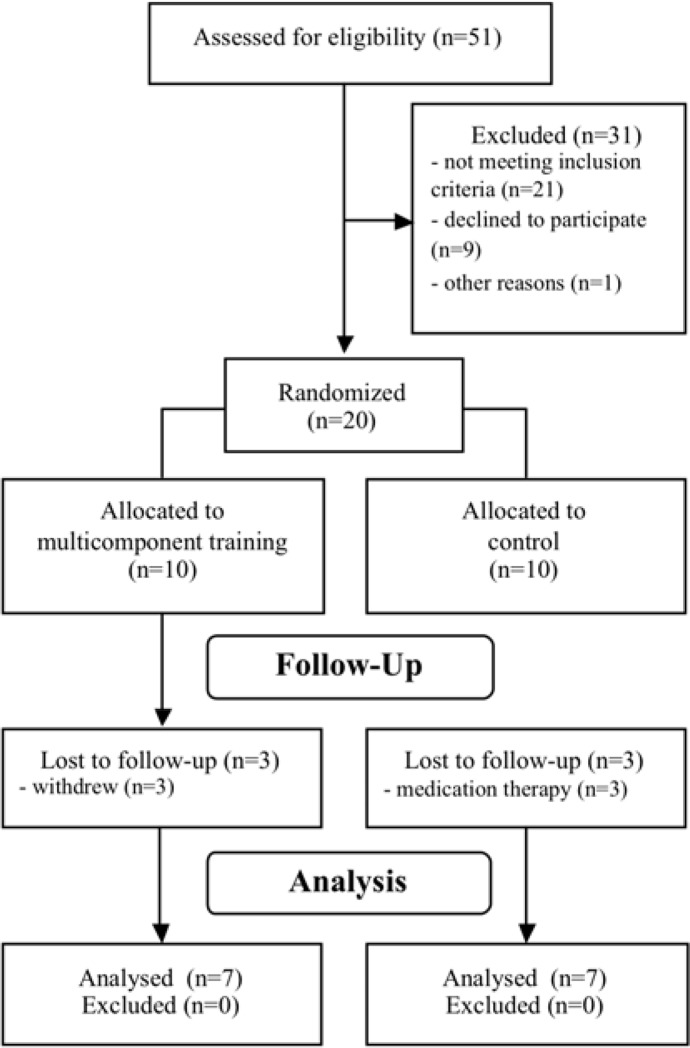
A summary of the recruitment strategy and allocation

Twenty postmenopausal women (aged 61.3 ± 6.0 years) with pre-diabetes and T2DM from João Pessoa City (Paraíba, Brazil) were randomly assigned into a community-based MT group (MTG, n=10), or a control group (CON, n=10). All participants were pre-diabetics or T2DM diagnosed in the last six months (American Diabetes Association, 2013). Exclusion criteria were (1) smokers; (2) systematic engagement in regular exercise of moderate-to-vigorous intensities for 20 minutes or more at least twice a week in the past 2 years; (3) history of stroke and/or myocardial infarction; (4) or any serious medical condition that prevented participants from adhering to the protocol or exercising safely. In addition, participants were excluded if they had taken medication such as bisphosphonates, raloxifene, hormone replacement, or glucocorticoids, during the study period or 12 months prior to the baseline due to the potential impact of these agents on bone metabolism. A summary of the recruitment strategy and allocation is presented in Figure 1.

Written informed consent was obtained from all participants. The study was approved by the ethics committee for health research as required by Resolution 196 of the National Health Council, 1996 Under the Protocol 94/06/07, already approved by the Center for Health Sciences UFPB.

### Procedures

Participants were tested on two occasions, prior to the beginning of training and after 8 months of training. Body fat percentage, fat-free mass and BMD for the femoral neck, greater trochanter, Ward’s triangle, total hip and whole body were measured by dual-energy X-ray absorptiometry (DEXA) using a Lunar DPX-L model (GE-Lunar Corp., Madison, WI). Total-body scans were taken using the same DEXA device. All scans were performed by the same technician using standard procedures as described in the Lunar user’s manual.

### Community-based exercise program

The community-based exercise program was composed of MT and was planned to include moderate-to-vigorous intensity (perceived exertion of 12–15 points on the Borg scale of 6–20 (Borg, 1982)). The MT included one aerobic exercise session (Mondays) involving 40 min walking; one weight-bearing exercise session (Wednesdays) performed with dumbbells (2–3 kg) and ankle weights (2–3 kg) in a circuit, including the main muscle groups (6 exercises, 3 sets, 15–20 repetitions); and one aquatic exercise session (in a swimming pool 25 m long and 1.40 m deep) consisting of static stretching exercises (4 exercises, 3 sets, 10 s) and muscular endurance exercises with water dumbbells and water resistance (4 exercises, 3 sets, 15–20 repetitions) for the major muscle groups.

The average adherence rate to all training sessions was 85%, however, the participants were informed that a minimum of 77 sessions during the 32 weeks (80% compliance) was required to be included in the analysis.

### Statistical analysis

The Shapiro-Wilk test was used to test the normality of the data. The Wilcoxon test was used for within-group comparisons. Between-group comparisons of difference percent changes were performed using the Mann Whitney U test. All data were analyzed using the statistical software IBM SPSS Statistics for Macintosh, version 19.0.0 (SPSS Inc., Chicago, IL), and the level of significance was set at p < 0.05.

## Results

Body composition and BMD variables, in both groups, at baseline and after the experimental period are shown in Table 1.

There was no significant difference between the groups in any variable at pre-test and post-test. However, the Wilcoxon scores identified significant changes in fat-free mass for both groups (pre vs. post-test). For the MTG there was a significant increase in fat-free mass after 32 weeks (p=0.018), while in the CON a significant decrease (p=0.028) was noticed. There were no changes in the body fat content.

The Wilcoxon scores also identified significant changes in Ward’s triangle (p=0.043) in the MTG. There were no changes in BMD for the femoral neck, greater trochanter, total hip and whole body.

## Discussion

The main finding of this study was that 8 months of a community-based MT program, significantly increased Ward’s triangle BMD (+7.8%), and fat-free mass (+4%) of elderly postmenopausal women with pre-diabetes and T2DM.

It is notable that MT had a greater effect on Ward’s triangle than it did on the femoral neck. Ward’s triangle is not a true anatomic area but is generated by the DEXA scan as the area having the lowest BMD in the femoral head. In a recent study focused on Ward’s triangle BMD, inverse correlation was found between Ward’s triangle BMD and age in women (Hayashida et al., 2012). It seems that Ward’s triangle area is the most suitable part to measure decreasing BMD with aging (Hayashida et al., 2012), and therefore, an increase in its density represents an important prevention factor for future osteoporotic fractures. The area of Ward’s triangle consists of trabecular bone which is comprised of a porous lattice structure with relatively low variable density, which provides flexibility and resilience to the overall bone structure. These properties allow the ability to absorb energy from impacts. The femur and greater trochanter are cortical bone formation with a more rigid structure, and probably more impact is necessary to change this structure (Verhulp et al., 2008; Holzer et al., 2009).

Aquatic exercises are more appropriate for subjects with chronic walking and balance problems, pain or advanced sarcopenia, but less effective in promoting an osteogenic response in the elderly (Gomez-Cabello et al., 2012). However, it seems that an addition of walking and weight-bearing exercises with free weights resulted in significant BMD improvements, as previously reported by Marques et al. (2011). To our knowledge, this is the first study with positive effects on postmenopausal women BMD after MT combining walking, resistance and aquatic exercises. Our data regarding the applied loading to the skeleton with the specific weight-bearing exercises suggest that moderate-impact forces result in significant changes in BMD, but only at Ward’s triangle.

Our data also indicate that an 8-month MT program, which combines resistance and aerobic training performed either on land or in water, induces favorable body composition adaptations, especially in the levels of fat-free mass (with a 4% increase, unlike the CON that lost 4.6%). To our knowledge, only few studies have demonstrated favorable effects of land and water-based training on body composition (Sousa et al., 2013; Stewart et al., 2005), but none in the levels of fat-free mass. This significant loss in fat-free mass in the CON may represent a major risk for all causes of mortality (Rantanen et al., 2000). In contrast, the improvements observed in the MTG may enhance metabolic syndrome health status, and may positively influence the BMD. In a recent study, Namwongprom et al. (2013) also concluded that fat-free mass had a significant beneficial effect on BMD in postmenopausal women.

A limitation of this study is that diet was not controlled. To minimize any confounding effects associated with variation in diet, the participants were informed to keep their dietary habits throughout the study period.

## Conclusions

On the basis of the results of the present study, it may be concluded that MT is effective in preventing osteoporosis and sarcopenia among postmenopausal women with pre-diabetes and T2DM. Another key point of the present research is the importance of low cost community-based exercise programs on health promotion and prevention of chronic diseases, contributing to political, economic and social development.

Although MT appears to be safe, postmenopausal women, particularly those with T2DM, should be carefully screened and supervised prior to and during exercise participation to ensure safety and injury prevention.

## Figures and Tables

**Table 1:** Pre- and post-training values and changes of body fat, fat-free mass, and bone mineral density for femoral neck, greater trochanter, ward’s triangle, total hip and whole body, over experimental protocol for both groups.

	CON (n = 7)				MTG (n = 7)			

	Pre	Post	Δ%	*p*	Pre	Post	Δ%	*P*
BF (%)	26.05 ± 6.5	26.25 ± 7.00	0,72	0.202	25,39±4.98	24.87 ± 5.16	−2,0	0.059
FFM (kg)	38.88 ± 6.1	37.18 ± 5.71	−4.4	0.007	37.77 ± 6.57	39.290 ± 5.96	4,0	0.005
FN (g/cm^2^)	0.76 ± 0.07	0.76 ± 0.08	0,0	0.646	0.81 ± 0.11	0.84 ± 0.11	3,7	0.092
GT (g/cm^2^)	0.72 ± 0.07	0.71 ± 0.06	−1,4	0.332	0.68 ± 0.06	0.69 ± 0.08	1.5	0.329
WT (g/cm^2^)	0.64 ± 0.08	0.62 ± 0.10	−2,8	1.000	0.67 ± 0.12	0.72 ± 0.14	7.5	0.009
TH (g/cm^2^)	1.05 ± 0.12	1.14 ± 0.45	8.6	0.063	1.07 ± 0.16	1.07 ± 0.18	0.0	0.499
WB (g/cm^2^)	0.78 ± 0.07	0.77 ± 0.09	−1.3	0.380	0.78 ± 0.15	0.80 ± 0.17	2.6	0.178

Values are mean ± S.E. CON: control group; MEG: multicomponent training exercise group; BF: body fat; FFM: fat-free mass; FN: femoral neck; GT: greater trochanter; WT: ward’s triangle; TH: total hip; WB: whole body. Δ% = [(post-training–pre-training)/pre-training]*100. p = values for pre-training vs. post-training
